
JOURNAL INFORMATION
==============================
NLM Title Abbreviation: Brain Behav
Journal ID: Brain Behav
NLM Journal ID: 101570837
Journal ID: BRB3

Brain and Behavior

EISSN: 2162-3279
Publisher: Wiley

ARTICLE INFORMATION
==============================
PMCID: PMC8518557
DOI: 10.1002/brb3.1688
Article ID: BRB31688
Article version: 1
Subjects: Issue Information, Issue Information

Issue Information

Electronic publication date: 2021 Sep 15
Publication date: 2021 Sep
Volume: 11
Issue: 9
Electronic Location ID: e1688
Copyright: © 2021 The Authors. Brain and Behavior published by Wiley Periodicals LLC
License: This is an open access article under the terms of the http://creativecommons.org/licenses/by/4.0/ License, which permits use, distribution and reproduction in any medium, provided the original work is properly cited.
License URL: https://creativecommons.org/licenses/by/4.0/

Figure count: 0
Table count: 0
Page count: 3

source-schema-version-number: 2.0
cover-date: September 2021
details-of-publishers-convertor: Converter:WILEY_ML3GV2_TO_JATSPMC version:6.0.8 mode:remove_FC converted:15.10.2021

==============================

Volume 11, Number 9

### Brain and Behavior

Open Access

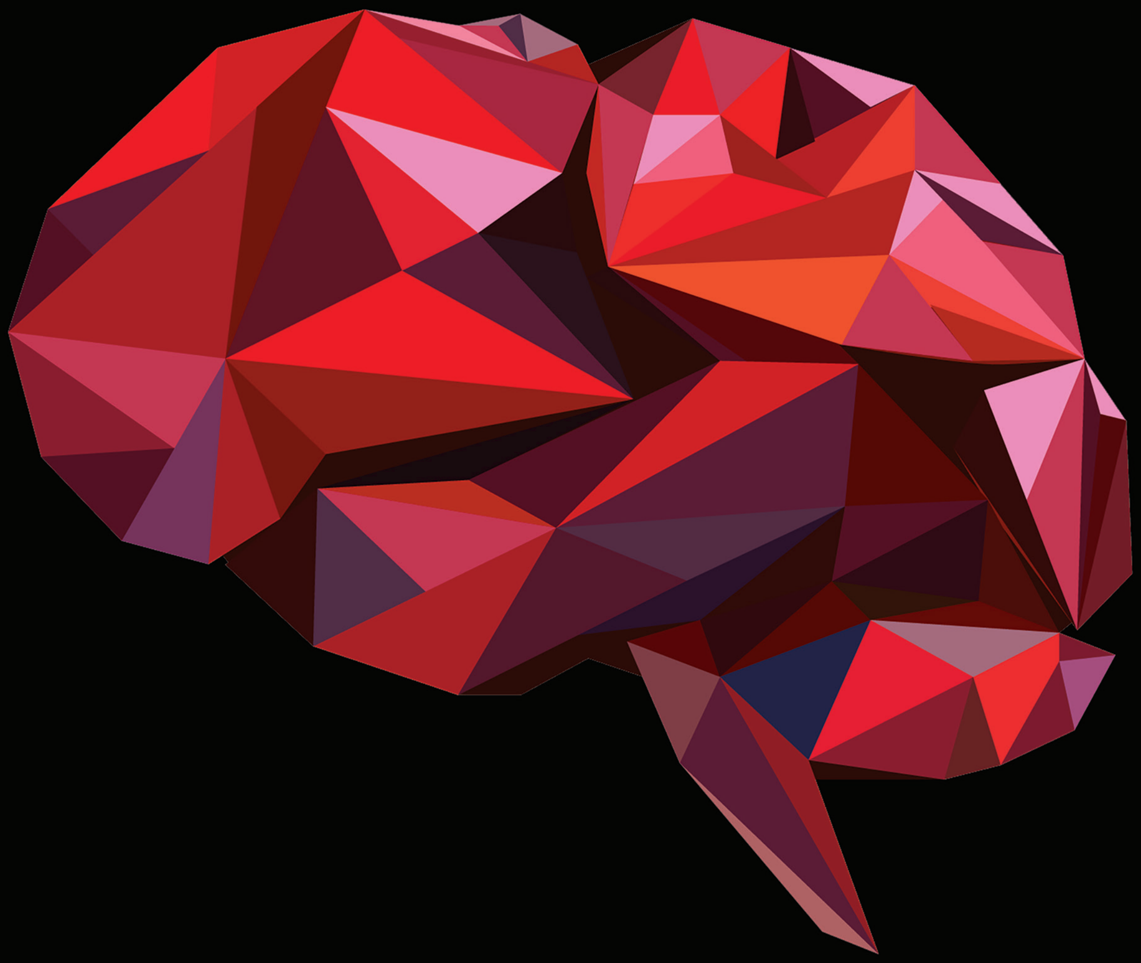

Editor-in-Chief
Nutan Sharma

WILEY

Brain and Behavior

Open Access

### Open Access and Copyright

All articles published by *Brain and Behavior* are fully open access: immediately freely available to read, download and share. All articles accepted from 14 August 2012 are published under the terms of the Creative Commons Attribution License. All articles accepted before this date were published under a Creative Commons Attribution Non-Commercial License. The Creative Commons Attribution License permits use, distribution and reproduction in any medium, provided the original work is properly cited and allows the commercial use of published articles.

Copyright on any research article in a journal published by *Brain and Behavior* is retained by the author(s). Authors grant Wiley a license to publish the article and identify itself as the original publisher. Authors also grant any third party the right to use the article freely as long as its integrity is maintained and its original authors, citation details and publisher are identified. Further information about open access license and copyright can be found at http://www.wileyopenaccess.com/details/content/12f25db4c87/Copyright–License.html.

### Disclaimer

The Publisher and Editors cannot be held responsible for errors or any consequences arising from the use of information contained in this journal; the views and opinions expressed do not necessarily reflect those of the Publisher and Editors, neither does the publication of advertisements constitute any endorsements by the Publisher and Editors of the products advertised.

Wiley Open Access articles posted to repositories or websites are without warranty from Wiley of any kind, either express or implied, including, but not limited to, warranties of merchantability, fitness for a particular purpose, or non-infringement. To the fullest extent permitted by law Wiley disclaims all liability for any loss or damage arising out of, or in connection with, the use of or inability to use the content.

For submission instructions, subscription, and all the latest information, visit wileyonlinelibrary.com/journal/brb3

*Cover image © aodaodaodaod / Shutterstock*

### Editor-in-Chief

Nutan Sharma
*Harvard Medical School and Massachusetts General Hospital*
*Boston, MA, USA*
*Address correspondence to the Editorial Office: brain@wiley.com*

### Senior Editors

David Belin
*University of Cambridge*
*Cambridge, UK*

Amanda Bischoff-Grethe
*University of California, San Diego*
*San Diego, CA, USA*

Bingren Hu
*University of Maryland School of Medicine*
*College Park, MD, USA*

Weiwen Wang
*Institute of Psychology, Chinese Academy of Sciences,*
*Beijing, China*

### EDITORIAL BOARD

Andrei Alexandrov
*Editor-in-Chief: 2011–2015*
*The University of Tennessee*
*Knoxville, TN, USA*

Hubert Amrein
*Texas A&M*
*College Station, TX, USA*

Isabelle Aubert
*University of Toronto*
*Toronto, Canada*

Sandra Black
*University of Toronto*
*Toronto, Canada*

Sandra Bosacki
*Brock University*
*St. Catharines, Canada*

Robert Bryan
*Baylor College of Medicine*
*Houston, TX, USA*

Alexander Bystritsky
*University of California,*
*Los Angeles*
*Los Angeles, CA, USA*

Hans Crombag
*University of Sussex*
*East Sussex, UK*

Mirella Dapretto
*University of California,*
*Los Angeles*
*Los Angeles, CA, USA*

David Diamond
*University of South Florida*
*Tampa, FL, USA*

Ilona Grunwald-Kadow
*Max Planck Institute of*
*Neurobiology*
*Planegg, Germany*

Martin Hagger
*Curtin University*
*Perth, Australia*

Jozsef Haller
*Hungarian Academy of Sciences*
*Budapest, Hungary*

Ahmad Hariri
*Hungarian Academy of Sciences*
*Budapest, Hungary*

Shu-Leong Ho
*University of Hong Kong*
*Pokfulam, Hong Kong*

Andrea Huber Brösamle
*Helmholtz Center Munich*
*Munich, Germany*

Kazuhiro Ikenaka
*National Institute for*
*Physiological Sciences*
*Aichi, Japan*

Stephen Jackson
*University of Nottingham and*
*Korea University*
*Nottingham, UK*

Takeshi Kaneko
*Kyoto University*
*Kyoto, Japan*

Kenneth Kosik
*University of California,*
*Santa Barbara*
*Santa Barbara, CA, USA*

Klas Kullander
*Uppsala University*
*Uppsala, Sweden*

Jonathan Lee
*University of Birmingham*
*Birmingham, UK*

Edward Levin
*Duke University*
*Durham, NC, USA*

Bai Lu
*Tsinghua University*
*Beijing, China*

Randolph Marshall
*Columbia University Medical*
*Center*
*New York, NY, USA*

Maryann Martone
*Editor-in-Chief: 2015–2018*
*University of California,*
*San Diego*
*San Diego, CA, USA*

Cheryl McCormick
*Brock University*
*St. Catharines, Canada*

Douglas Mennin
*Hunter College*
*New York, NY, USA*

Martin M. Mortazavi
*California Neurological Institute*
*Santa Barbara, CA, USA*

Declan Murphy
*King's College London*
*London, UK*

Heinz Reichmann
*University of Dresden*
*Dresden, Germany*

Richard Rende
*Brown University*
*Providence, RI, USA*

Giacomo Rizzolatti
*University of Parma*
*Parma, Italy*

Fred Sabb
*University of California,*
*Los Angeles*
*Los Angeles, CA, USA*

Veronika Skvortsova
*Russian State Medical University*
*Moscow, Russia*

Kai-Christian Sonntag
*Harvard Medical School and*
*McLean Hospital*
*Boston, MA, USA*

Takashi Suzuki
*Tokyo Institute of Technology*
*Tokyo, Japan*

Wai Kwong Tang
*Chinese University of Hong Kong*
*Ma Liu Shui, Hong Kong*

Wesley Thompson
*University of California,*
*San Diego*
*San Diego, CA, USA*

Tanya N. Turan
*Medical University of South*
*Carolina*
*Charleston, SC, USA*

Hartmut Wekerle
*Max Planck Institute of*
*Neurobiology*
*Planegg, Germany*

Jianhong Zhu
*Fudan University*
*Shanghai, China*
